# Iron deficient erythropoiesis might play key role in development of anemia in cancer patients

**DOI:** 10.18632/oncotarget.5658

**Published:** 2015-10-26

**Authors:** Silvia Park, Chul Won Jung, Kihyun Kim, Seok Jin Kim, Won Seog Kim, Jun Ho Jang

**Affiliations:** ^1^ Division of Hematology-Oncology, Department of Medicine, Samsung Medical Center, Sungkyunkwan University School of Medicine, Seoul, Korea

**Keywords:** cancer, anemia, soluble transferrin receptor, hepcidin

## Abstract

**Introduction:**

Multifactorial pathogenesis is involved in anemia of cancer patients and defining the causes of anemia is not always simple.

**Methods:**

The incidence of anemia among 4 major cancers (gastric, colorectal, lung cancer and hepatocellular carcinoma), and biochemical features of anemia using ferritin, CRP, hepcidin and soluble transferrin receptor (sTfR) were assessed. Anemia was defined either by hemoglobin (Hb) ≤11 g/dL or a drop of Hb 2 g/dL or more during anticancer treatment.

**Results:**

Among the 345 patients including 152 lung cancer, 101 gastric cancer, 69 colorectal cancer and 23 hepatocellular carcinoma, 49 patients (14.2%) had anemia at their initial diagnosis of cancer. During treatment, 129 (37.4%) experienced anemia, and 34 (26.4%) were treated mostly by transfusion. Biochemical feature of anemia was examined with 39 patients' samples. When comparing to the reference value from general population, cancer patients showed numerically higher ferritin, sTfR, CRP and hepcidin level. Among the cancer patients, anemic patients had significantly higher ferritin (*p* = 0.050) and sTfR (*p* = 0.009) level compared to non-anemic patients.

**Conclusion:**

Anemia is a common issue in cancer patients and is largely undertreated with sub-optimal diagnoses of cause. The rates of anemia increase significantly during anti-cancer treatment and appear to be largely associated with iron deficiency.

## INTRODUCTION

The incidence of anemia in cancer patients is frequent although with large variability in reported studies ranging from 30 to 90% [1]. Anemia is important in cancer patients as it may further weaken this already fragile population [2] and has been associated with a poorer clinical outcome [3, 4]. Therefore, it is recognized that management and correction of anemia is important. Unfortunately appropriate management is not always initiated and is probably due to the gravity of the underlying malignancy [5]. To aid in having treatment of anemia as routine practice, it is important to understand the frequency and clinical impact but also to enable identification of the underlying cause in combination with bio-markers that can guide beneficial treatment.

Anemia in patients with cancer is understood to have a multifactorial pathogenesis [6–11] with the vast majority believed to have some degree anemia of chronic disease (ACD) secondary to their cancer [12], of which functional iron deficiency (FID) plays a key role [13, 14]. Insufficient iron incorporation into erythroid precursor despite of apparently adequate body iron stores is a major phenomenon of FID [14] and seems to be more frequent than absolute iron deficiency in cancer patients probably due to the underlying inflammation from malignancy [15, 16]. In situations involving bleeding (including from the cancer), absolute iron deficiency can be the major cause of anemia [17] although absolute iron deficiency and functional iron deficiency from ACD can occur simultaneously [18]. Moreover, bone marrow (BM) suppression either by malignant cell infiltration [19] or myelosuppressive chemotherapy [7] as well as nutritional deficiencies associated with appetite loss [20] can put a strain on an already anemic cancer patient. Overall, ACD and chemotherapy induced anemia (CIA) are the most likely causes of anemia [2]. However, due to the potentially multifactorial complexity of anemia, defining the causes in cancer patients is not always simple, and conventional evaluation has only limited value [21].

Currently, National Comprehensive Cancer Network (NCCN) provides guideline for cancer- and chemotherapy induced anemia diagnosis and management [22]. Evaluation of anemia within the guideline broadly includes mean corpuscular volume (MCV), reticulocyte index, iron profile, vitamin B12, folate, evaluation for hemorrhage and hemolysis etc. and is not different from that used within the general population despite the particularities of anemia in cancer patients. For example, iron, total iron binding capacity (TIBC) and ferritin are being identically suggested when evaluating iron deficiency anemia although cancer patients are expected to be exposed to inflammatory condition in some degree, which can elevate ferritin level and potentially obscure the matter of iron deficiency [14]. Moreover, TIBC that has positive correlation with transferrin [23] may be decreased in poorly nourished patients [24], which eventually leads increased transferrin saturation ratio [25]. While there is an additional guide for functional iron deficiency, it is only being considered in patients receiving erythropoiesis stimulating agents (ESAs) although functional iron deficiency is not the problem only in these individuals. As more relevant biomarkers of anemia, hepcidin, the master regulator of iron homeostasis, contributes to the development of anemia of chronic disease (ACD) [26, 27]. Additionally, soluble transferrin receptor (sTfR) is a good indicator of absolute iron deficiency and/or iron restricted erythropoiesis in patients with inflammation [28–30].

Currently, treatment options for anemia in cancer patients include red blood cell (RBC) transfusion, ESA, and iron supplementation, with each having considerable pros and cons [22]. RBC transfusion is the only option providing immediate correction of anemia but is associated with number of risks such as infection and iron overload [31, 32] as well as potential recurrence of cancer [33]. Treatment with ESA has been shown to decrease RBC transfusion [34–36], but concerns on increased mortality and tumor progression limited its use in curative setting [37, 38]. Although intravenous iron supplementation improves the effectiveness of ESA in correcting anemia [39, 40], it still has limited data as a monotherapy [41]. Conversely, oral iron has not demonstrated benefits when used in combination with an ESA [42] or as a monotherapy [43] in this patient population. Consequently, optimal management for anemia remains controversial in cancer patients [5].

This study assesses anemic patients with cancer primarily from a clinical aspect with a secondary-analysis from a biochemical aspect. For the clinical aspect, our objective was to evaluate the incidence of anemia and then describe the management of these patients in routine clinical care. For the biochemical aspect, we attempt to define the characteristics of anemia in cancer patients via comparison of more sensitive parameters including sTfR and hepcidin and compare these for anemic and non-anemic patients with cancer.

## RESULTS

### Anemia at initial diagnosis of cancer

A total of 345 patients were analyzed, and their baseline characteristics are documented in Supplementary Table 1. The median age was 59 (26–84) years, and male patients were more dominate (*n* = 216, 62.6% for men, *n* = 129, 37.4% for women). Lung cancer was the most common (*n* = 152, 44.1%) followed by gastric cancer (*n* = 101, 29.3%), colorectal cancer (*n* = 69, 20.0%) and hepatocellular carcinoma (*n* = 23, 6.7%). Overall, 49 patients (14.2%) had anemia at their initial diagnosis of cancer (Table 1). According to the diagnosis, anemia was initially observed in 20.3% of patients with gastric cancer, 13.9% with colorectal cancer, 8.7% with hepatocellular carcinoma (HCC) and 12.5% with lung cancer (*p* = 0.421). There were no significant differences in the proportion of anemia at initial diagnosis between patients with non-metastatic disease (14.8%) and metastatic disease (13.6%) (*p* = 0.787). The mean value of baseline hemoglobin level was 13.2 g/dL, and MCV level was 89.7 fL. Female patients had lower baseline Hb level compared to male patients (13.9 g/dL for men and 12.6 g/dL for women, *p* < 0.001), and lower MCV value (90.7 fL for men and 89.5 fL for women, *p* = 0.005). According to cancer sites, HCC showed highest baseline hemoglobin level (14.0 g/dL). The median value of MCV was also highest in HCC (93.5 fL), and no patients with HCC showed MCV level lower than 80 fL (range, 85.4–104.3 fL). This differed to patients with other types of cancer with the lowest MCV values of 48.5 fL, 66.0 fL, and 73.6 fL for gastric cancer, colorectal cancer and lung cancer, respectively. Regarding overall morphologic feature of anemia, normocytic anemia was most common (37/49, 75.5%), microcytic anemia and macrocytic anemia comprised 20.4% (10/49) and 4.1% (2/49), respectively. Relative frequency (%) of morphologic type of anemia showed different distribution by diagnosis. The proportion of microcytic, normocytic and macrocytic anemia was 21.4%, 71.4% and 7.1% for gastric cancer; 42.9%, 57.1% and 0% for colorectal cancer; 0%, 100%, 0% for HCC; 5.3%, 89.5% and 5.3% for lung cancer).

**Table 1:** The incidence of anemia at diagnosis and comparison by clinical characteristics

		Hemoglobin (g/dL)		MCV (fL)		Anemia	
		mean ± SD or median (IQR, range)	*p*-value	mean ± SD or median (IQR, range)	*p*-value	number (%)	*p*-value
Overall		13.2 ± 2.0		89.7 ± 6.8		49 (14.2%)	
Comparison by clinical characteristics							
Age	<60 ≥60	13.4 (2.6, 7.0–17.4) 13.1 (2.2, 8.1–18.4)	0.085	89.7 (6.2, 48.5–104.3) 90.8 (5.8, 32.0–108.1)	**0.001**	25 (14.2) 24 (14.2)	0.999
Gender	male female	13.9 (2.8, 7.6–18.4) 12.6 (2.0, 7.0–15.8)	**<0.001**	90.7 (5.9, 48.5–108.1) 89.5 (5.2, 32.0–106.9)	**0.050**	28 (13.0) 21 (16.3)	0.393
Cancer sites	Gastric cancer Colorectal cancer Hepatocellular carcinoma Lung cancer	13.8 (3.2, 7.0–18.4) 13.5 (3.0, 7.6–17.4) 14.0 (2.4, 9.4–16.5) 13.0 (2.0, 7.7–16.3)	**0.013**	90.4 (6.3, 48.5–106.9) 88.8 (7.8, 66.0–98.6) 93.5 (4.8, 85.4–104.3) 90.4 (4.4, **73.6**–108.1)	**<0.001**	14 (20.3) 14 (13.9) 2 (8.7) 19 (12.5)	0.421
Stage	Non-metastatic disease Metastatic disease	13.4 (2.7, 7.0–18.4) 13.0 (2.5, 7.7–17.4)	0.073	90.6 (5.2, 32.0–103.8) 89.9 (5.9, 67.2–108.1)	0.423	28 (14.8%) 21 (13.6%)	0.787

Abbreviates: SD, standard deviation; IQR, interquartile range

### Anemia during anti-cancer treatment and treatment for the anemia

Patients were treated with chemotherapy (*n* = 215, 62.3%), chemo-radiation (*n* = 75, 21.7%) and target agents (*n* = 55, 15.9%) as 1^st^ line anti-cancer treatment (Supplementary Table 1). The distribution of treatment according to the treatment intent was adjuvant (*n* = 170, 49.3%), palliative (*n* = 154, 44.6%), neoadjuvant (*n* = 6, 1.7%) and definitive (*n* = 15, 4.3%). During treatment, 129 among 345 patients (37.4%) experienced anemia (Table 2). According to clinical features, the elderly were more likely to be exposed to anemia during treatment (31.8% for <60 years and 43.2% for ≥60 years, *p* = 0.029), and lung cancer was the type of cancer causing anemia most frequently (39.6% for gastric cancer; 21.7% for colorectal cancer; 26.1% for HCC; 44.7% for lung cancer, *p* = 0.007). Although there was no statistical significance, patients who were treated with chemo-radiation had a numerically higher frequency of anemia (44.0%) compared to chemotherapy alone (37.7%) or target agents (27.3%) (*p* = 0.060). Among the 129 patients with anemia, 34 patients (26.4%) were treated for their anemia, almost by RBC transfusion (RBC transfusion, *n* = 28; oral iron replacement, *n* = 5; ESA, *n* = 1).

**Table 2:** The incidence of anemia during treatment and comparison by clinical characteristics

**Anemia during 1^st^ line anti-cancer treatment**			
**Overall frequency**		129/345 (37.4%)	
Morphologic features of anemia (*n* = 129)	Microcytic anemia Normocytic anemia Macrocytic anemia	1 (0.8%) 124 (96.1%) 4 (3.1%)	
Comparison by clinical characteristics		Anemia, number (%)	*p*-value
Age	<60 ≥60	56 (31.8) 73 (43.2)	**0.029**
Gender	male female	77 (35.6) 52 (40.3)	0.387
Cancer sites	Gastric cancer Colorectal cancer Hepatocellular carcinoma Lung cancer	40 (39.6) 15 (21.7) 6 (26.1) 68 (44.7)	**0.007**
Stage	Non-metastatic disease Metastatic disease	67 (35.1) 62 (40.3)	0.323
Treatment	Chemotherapy Chemo-radiation Target agents	81 (37.7) 33 (44.0) 15 (27.3)	0.060

### Biochemical parameters in cancer patients

Supplementary Table 2 shows the baseline characteristics of *the* 39 patients whose serum samples were analyzed. There were 27 male (69.2%) and 12 female patients (30.8%), and the median age was 58 years (range, 27–82). The distribution of the diseases was as follows; gastric cancer (*n* = 11, 28.2%), biliary tract cancer (*n* = 6, 15.4%), lung cancer (*n* = 18, 46.2%) and lymphoma (*n* = 4, 10.3%). At the time of sample collection, 31 patients (88.6%) had metastatic disease and 26 (66.7%) experienced chemotherapy within 6 months. The median hemoglobin level was 9.5 g/dL and the mean MCV value was 92.2 fL. For other biochemical parameters, the mean ferritin level was 240.7 ng/mL in men and 224.7 ng/mL in women (Supplementary Table 3). The mean level of sTfR, CRP, and hepcidin were 1.03 ug/mL, 14.73 mg/L, and 12.50 ng/mL, respectively. When comparing to reference range from *the* general population, 39 cancer patients in this study showed numerically higher ferritin, sTfR, CRP, and hepcidin level.

### Comparison of cancer anemia related parameters between anemic vs non-anemic patients

Among 39 patients with samples, 21 patients (53.8%) *had* Hb ≤11 g/dL at the time of serum collection (Table 3). There were no significant differences in clinical characteristics in terms of age, gender, history of chemotherapy exposure within 6 months and the presence of metastatic disease between the anemic and non-anemic patients. Among the cancer patients, anemic patients showed significantly higher ferritin and sTfR level compared to non-anemic patients (166.7 ng/mL vs. 295.0 ng/mL for ferritin, *p* = 0.050; 0.69 ug/mL vs.1.28 ug/mL for sTfR, *p* = 0.009). Although not statistically significant, the levels of CRP and hepcidin were more elevated in anemic patients. Each cancer anemia related parameter was also analyzed for defining correlation between hemoglobin level (Table 4). Among *these* parameters, sTfR showed significant negative correlation with hemoglobin level (*p* = 0.013, γ = − 0.396).

**Table 3:** Comparison of biochemical parameters between anemic vs non-anemic patients

			No anemia (*n* = 18)	Anemia (*n* = 21)	*p*-value
Clinical characteristics	Age, years	Median (IQR, range)	56 (16, 38–73)	60 (12, 27–82)	0.144
	Gender	Male Female	12 (66.7%) 6 (33.3%)	15 (71.4%) 6 (28.6%)	0.748
	Chemotherapy within 6 months	Yes No	11 (61.1%) 7 (38.9%)	15 (71.4%) 6 (28.6%)	0.496
	Metastasis*	Yes No	14 (87.5%) 2 (12.5%)	17 (89.5%) 2 (10.5%)	1.000
	MCV, fL		92.06 ± 0.81	92.27 ± 2.29	0.932
Biochemical parameters	Ferritin, ng/mL	Median (IQR)	166.7 ± 29.7	295.0 ± 55.5	**0.050**
	sTfR, ug/mL	Mean ± SD	0.69 (0.49)	1.28 (0.77)	**0.009**
	CRP, mg/L	Median (IQR)	1.55 (5.89)	4.95 (33.19)	0.055
	Hepcidin, ng/mL	Median (IQR)	7.05 (8.91)	12.99 (22.61)	0.324

Abbreviates: SD, standard deviation; IQR, interquartile range

* Lymphoma patients (*n* = 4) were excluded from the analysis

**Table 4:** Correlation of each parameter and clinical characteristics

		Ferritin		sTfR		CRP		Hepcidin	
		r	*p*-value	r	*p*-value	r	*p*-value	r	*p*-value
Clinical features at the time of sample collection	Age	0.443	**0.005**	–	0.122	–	0.145	0.389	**0.014**
	Gender	–	0.454	–	0.751	–	0.881	–	0.800
	Chemo	0.382	**0.016**	–	0.849	–	0.115	0.498	**0.001**
	Metastasis	–	0.069	–	0.703	0.338	**0.047**	0.356	**0.036**
	Hemoglobin	–	0.749	−0.396	**0.013**	–	0.164	–	0.613
	MCV	0.503	**0.001**	–	0.552	–	0.583	0.422	**0.007**
Anemia related biochemical parameters	Ferritin								
	sTfR	–	0.829						
	CRP	0.342	**0.033**	–	0.482				
	Hepcidin	0.876	**< 0.001**	–	0.684	0.350	**0.029**		

### Correlation of each parameter and clinical characteristics

Each cancer anemia related parameter and clinical characteristics were analyzed for defining correlation (Table 4). Older age and higher level of ferritin and hepcidin were correlated (*p* = 0.005, *γ* = 0.443 for ferritin; *p* = 0.014, *γ* = 0.389 for hepcidin). None of the cancer anemia related parameters had correlation with gender. Exposure to chemotherapy within 6 months was positively correlated to ferritin (*p* = 0.016, *γ* = 0.382) and hepcidin (*p* = 0.001, *γ* = 0.498). Patients with metastatic disease had significantly higher CRP (*p* = 0.047, *γ* = 0.338) and hepcidin (*p* = 0.036, *γ* = 0.356) level. Between cancer anemia related parameters, CRP, ferritin and hepcidin were positively correlated with each other (*p* = 0.033, *γ* = 0.342 for CRP and ferritin; *p* < 0.001, *γ* = 0.876 for ferritin and hepcidin; *p* = 0.029, *γ* = 0.350 for CRP and hepcidin).

## DISCUSSION

Anemia is a common problem in cancer patients globally with rates as high as 90% [44] although significant inconsistency for observed prevalence exists due to the varied definitions of anemia used in the studies [1]. A universal normal Hb value is difficult to define even among healthy subjects, and the expected normal value differs by gender. However, experts generally suggest that a Hb level of 11 g/dL should prompt an evaluation of anemia and a drop of 2 g/dL or more should arouse concern for assessment of anemia in patients with high baseline (or pre-treatment) Hb concentration [22]. We also used this definition in our study. Based on the above definition of anemia, the prevalence of anemia in the studied South Korean population of cancer patients was 14.2% at diagnosis and 37.4% during 1^st^ line anti-cancer treatment.

We analyzed patients representing the 4 most prevalent cancers affecting South Korean patients. Anemia was most frequently observed in gastric cancer at the time of diagnosis (20.3%). When anemia is more liberally defined as Hb <12 g/dL, the number of anemic patients increased to 41.6% in gastric cancer and 46.4% in colorectal cancer, showing high incidence of anemia at initial diagnosis of gastrointestinal tract cancer. Hepatocellular carcinoma is a type of cancer that can accompanied with paraneoplastic erythrocytosis [45]. Accordingly, although HCC in Korea is largely associated with hepatitis B virus infection related liver cirrhosis [46–48], which has potential risk of bleeding complication, anemia was not common in HCC in this analysis, and the median Hb level was also highest (14.0 g/dL) compared to other type of cancers. Interestingly, lung cancer was the cancer that resulted in the most cases of anemia during anti-cancer treatment (44.7%). Previous studies have already identified that the incidence of chemotherapy-induced anemia is high in lung cancer and gynecological malignancy [7, 49], probably due to frequent use of platinum-based regimen. Among the 152 lung cancer patients in this study, 120 patients (78.9%) were exposed to platinum based regimen as first line therapy, and 59 of 120 patients (49.1%) experienced anemia, whereas only 9 of 32 patients (28.1%) who had been treated with non-platinum regimen as 1^st^ line treatment (e.g. iressa) experienced anemia. We also observed that elderly patients were more likely to be anemic with anti-cancer treatment, emphasizing the more fragile characteristics in this population. Whilst guidelines recommend evaluation and treatment of anemia, assessment for anemia was rarely performed. Additionally, once anemia diagnosed, only 26.4% received anti-anemia treatment and this was almost exclusively limited to RBC transfusions. This suggests that anemia management in cancer patients is receiving insufficient attention and substantial improvements are feasible. It also tells us that anemia is inevitably outside the physician's interest in cancer patients, which is probably because treating malignancy is a major concern for medical oncologists.

In addition, we performed an exploratory examination of the biochemical features associated with the anemia in cancer patients. As representative parameters for iron deficiency, with or without iron restricted erythropoiesis, ferritin and sTfR were measured. In addition, as a marker of anemia of chronic disease, CRP and hepcidin which are induced and elevated in inflammatory condition [50], were assessed. As ferritin can be elevated with inflammation even in an iron deficient state [51] and the elevation of hepcidin level can be disturbed by iron deficiency [52], cautious interpretation was required when analyzing the results. We also assessed relationships between anemia and the patients' clinical features. Finally, to aid understand the relevance and impact of the cancer on these parameters, we analyzed and compared markers to the ‘general population’. From a simple comparison of measurements to reference value in general population, we found that cancer patients showed numerically higher ferritin, sTfR, CRP and hepcidin. This possibly implies that cancer patients are more likely to be exposed to inflammatory condition based on higher ferritin, CRP and hepcidin level, and more likely to encounter an iron restricted erythropoiesis based on higher sTfR level. Correlation between clinical characteristics and each parameter showed several interesting results as follows. Patients who received chemotherapy recently showed higher ferritin and hepcidin. Also, CRP and hepcidin had positive correlation with more advanced staged disease. Given that CRP, ferritin and hepcidin are induced in inflammatory condition, these results may suggest that advanced disease and recent chemotherapy promote inflammatory conditions. Actually, positive correlation between CRP and hepcidin was proved in the analysis. Not surprisingly, serum ferritin level correlated more strongly to CRP and hepcidin compared to sTfR in cancer patients. This reaffirms that ferritin level is closely linked to inflammatory condition and it cannot be a reliable marker of iron deficiency at least in cancer patients.

Based on aforementioned results, we tried to define the biochemical features of anemia in cancer patients via comparison of parameters between anemic and non-anemic patients. Having no significant difference in clinical characteristics between the two groups (anemic vs non-anemic), anemic patients showed higher ferritin (*p* = 0.050), sTfR (*p* = 0.011), CRP (*p* = 0.055), and hepcidin level (*p* = 0.324). Although CRP and hepcidin level were not significantly different between the groups, consistent increase of these two markers and significantly elevated ferritin level in anemic patients with cancer implies that anemia in cancer seems to be closely related to inflammatory process (ACD), which is in accordance with common belief. Of note, however, we can also identify that iron deficiency and/or iron restricted erythropoiesis also contributes considerably to pathogenesis of anemia in cancer patients. As sTfR levels are not increased in patients with inflammation, but are increased if the patients have concomitant true iron deficiency, we chose the sTfR as a reliable marker of the iron status in this study [28]. Moreover, sTfR has been reported to correlate well with tissue iron deficiency rather than storage iron depletion [53]. Given the complexities of anemia in cancer patients or indeed, the complexity of managing treatment of the primary cancer, appropriate treatment for anemia can be easily ignored. However, the lack of treatment may be placing our patients at increased risks and almost definitely will be negatively impacting the patients' quality of life. In this regard, observation in this study that anemic cancer patients more likely to have higher sTfR level has to be paid attention. It might suggest that some proportion of patients may benefit from iron replacement for their anemia although it can be easily overlooked in real practice. It is important to acknowledge the limitation with the above interpretations. As known, gastrointestinal cancers may have iron deficiency anemia due to chronic blood loss and therefore, the inclusion of gastric cancers in the biochemical data subset limits the strength of the previous conclusion points. Unfortunately this could not be avoided as only limited samples were available and hence we were obliged to use gastric cancer patients' samples as well as biliary tract cancer and lymphoma patients' samples (different diseases from those of clinical data set). In addition, the limited samples available for analysis resulted in a small sample size without appropriate statistical powering and further it is acknowledged that there is heterogeneous timing of the sample collection (i.e., before treatment, after treatment, etc). As such, careful interpretation prior to application to the clinical settings is required. However, these results warrant further investigation and confirmation of the findings in future observational and/or interventional studies.

In patients with chronic heart failure, data suggests that iron deficiency may be more prognostically important than anemia as an independent risk factor [54, 55]. Whilst similar correlations of iron deficiency with mortality have not been assessed in cancer patients, Ludwig et al (in a study of over 1500 patients) noted that iron deficiency was frequent in cancer and associated with advanced disease, close proximity to cancer therapy, and poor performance status [56]. Although further information is required to better assess the relative clinical impact of anemia and iron deficiency, especially when secondary to anti-cancer treatments, initial studies have demonstrated benefit when treating with IV iron in combination with ESA and, more recently, evidence is emerging to suggest a role for IV iron alone [41].

Whilst understanding the cause of the anemia is important, it is likely to be more important to understand the appropriate bio-markers that can permit management of these patients that results in clinical benefit. The combination of ferritin and transferrin saturation (TSAT) has been largely utilized as the routine parameters to assess iron deficiency. As such, additional measurements on these conventional iron parameters were assessed in patients where serum samples permitted (29 of 39 patients). Among the conventional iron parameters, serum iron was significantly lower in anemic patients (median 89.5 ug/dL vs 37.05 ug/dL, *p* = 0.024) (Supplementary Table 4–1). Both TIBC and TSAT were also lower in anemic patients although there was no statistical significance. When analyzing the correlation with other biochemical markers, serum iron was negatively correlated to CRP (*p* = 0.002, *γ* = −0.559), and TIBC showed inverse correlation with all inflammation related markers as follows: ferritin (*p* < 0.001, *γ* = −0.698), CRP (*p* = 0.006, *γ* = −0.497) and hepcidin (*p* < 0.001, *γ* = −0.654). As a derivative from these two markers (iron, TIBC), TSAT had significant correlation with CRP (*p* = 0.017, *γ* = −0.441) (Supplementary Table 4–2). These results suggest that conventional iron parameters including iron, ferritin, TIBC and TSAT are largely affected by cancer associated inflammation, thus seem to be less reliable for identifying patients who benefit from iron supplementation. Conversely, sTfR that decreases after adequate iron supplementation regardless of marrow iron stores appears to be a good indicator of functional iron deficiency [57], and might provide information on individual necessity for iron supplementation. However, the interpretation of an individual value may be tricky due to a lack of international standardized value and different reference ranges according to assays. For the hepcidin analyses, it has the same problem with sTfR. Currently there is lack of harmonization tool for standardization of the value and therefore interpretation of hepcidin levels (higher/lower than general population) has been determined using the reference range suggested by each kit's manufacturer, which remains major hurdle to the use of sTfR and hepcidin in a real practice [30].

In summary, anemia is a common issue in cancer patients and is largely undertreated with sub-optimal diagnoses of cause in South Korea. When treated, this is mostly with a blood transfusion. The rates of anemia increase significantly during treatment with anti-cancer therapies. Whilst limited samples were available for analysis, there appears to be further increased rates of anemia in cancer patients secondary to iron deficiency. Evidence on the risks of not treating and the emerging data demonstrating benefits of treating warrant a more systematic approach to early diagnosis both prior to commencing anti-cancer therapies as well as during treatment.

## MATERIALS AND METHODS

### Patients and data collection

There were two data subsets used in this analysis. The first was used for reporting the incidence of anemia and the management or treatment in routine clinical practice whilst the other was used to evaluate the biochemical features of anemia in cancer patients. Data for describing the incidence and treatment practices was collected from 4 major cancers in South Korea and included gastric cancer, colorectal cancer, lung cancer and hepatocellular carcinoma. A total of 345 patients were treated for aforementioned malignancies by the medical Oncology department at Samsung Medical Center between January and April 2012. These patients had been exposed to chemotherapy, radiotherapy, chemo-radiation, and/or target agents. Patients undergoing surgical resection only as anti-cancer treatment were excluded from this data collection and subsequent analysis. The second data was developed from stored serum samples that had been collected since July 2008. These serum samples had been obtained from patients providing informed consent and had been stored at −70°C in Samsung Medical Center. Thirty nine stored frozen serum samples from 4 types of cancer patients (gastric cancer, biliary tract cancer, lung cancer and lymphoma) were selected and used to analyze ferritin, solublue transferrin receptor (sTfR), CRP and hepcidin. Each parameter was analyzed using commercial ELISA kits as follows; DRG^®^ Ferritin ELISA (EIA-4408), DRG^®^ Human sTFR ELISA (EIA-4256), CRP (Human) ELISA kit (Abnova. No. KA0238), and DRG^®^ Hepcidin 25 ELISA (EIA-5258). Reference values or ranges provided by manufacturer were listed in Supplementary Table 3. In case of CRP, the level that exceeds 100 mg/L (in 1 patient) could not be evaluated its precise value and encoded as 100 mg/L for this analysis; the latest value under 100 mg/L was 95.13 mg/L.

### Definition and objectives

Anemia was defined using evaluation criteria per the expert recommendations of NCCN guidelines; which specifies anemia as a hemoglobin (Hb) concentration of 11 g/dL or less. In patients with a pre-treatment Hb concentration greater than 11 g/dL, anemia was defined by a Hb ≤ 11 g/dL during anti-cancer treatment or if a drop of Hb by 2 g/dL or more (from baseline) was observed. The type of anemia by morphologic feature was stratified according to mean corpuscular volume (MCV) value and was defined as follows; microcytic anemia when MCV < 80 fL; normocytic anemia when MCV 80–100 fL; and macrocytic anemia when MCV >100 fL. The primary objective was to estimate the incidence of anemia and describe the management of these patients among 4 major cancers in South Korea. Incidence of anemia was separately considered at 2 distinct time points; “at diagnosis” and “during anti-cancer treatment”. As an anti-cancer treatment, all treatment modalities but surgery were accounted, and only first line therapy was considered for minimizing complexities from the effect of subsequent treatment on anemia development. Secondary objectives included the incidence of anemia according to cancer type and treatment modality, assessment of morphologic type of anemia, and biochemical features of anemia. The selected biochemical parameters included serum ferritin, sTfR, CRP and hepcidin. These were titled as ‘cancer anemia related parameters' for convenience.

### Statistical analysis

Based on an initial estimation for anemia incidence of 50% (+/− 10%) and 95% C.I, at least 97 patients were required for the analysis. Based on our dataset, a total of 417 patients that were treated by medical oncologists from a planned period (between January and April 2012) were included. We subsequently excluded 9 patients with multiple primary cancers, 36 patients who lacked initial hemoglobin level (mainly due to transfer to our hospital after initiation of treatment) and 27 patients who could not finish their planned 1^st^ line anti-cancer treatment. Finally we analyzed 345 patients that met eligibility, so that we can analyze the incidence of anemia more precisely with 95% C.I of +/5.3%. We separately analyzed a second cohort of 29 patients to explore the difference of biochemical values involving ferritin, sTfR, CRP and hepcidin between anemic and non-anemic cancer patients. Numerical variables (hemoglobin, MCV, ferritin, CRP, sTfR, and hepcidin) are expressed as ‘mean ± standard deviation (SD)’ or ‘median (interquartile range, IQR). The incidence of anemia was presented using descriptive statistics. Differences between groups were calculated using the Chi square test or Fisher's exact test for categorical variables, and using the two sample *t*-test or Mann-Whitney test for continuous variables. If the comparison between more than two groups (cancer sites) was performed, one-way Anova or Kruskal-Wallis test was used for the continuous variables. Correlation between variables was examined by Pearson's correlation analysis or Spearman's correlation analysis. Statistical analysis was performed using the Statistical Package for the Social Sciences (SPSS) v.17.0 (SPSS Inc., Chicago, IL, USA).

## SUPPLEMENTARY TABLES


