# Supplementary material for: Iron deficient erythropoiesis might play key role in development of anemia in cancer patients
Source: Oncotarget. 2015 Oct 26;6(40):42803–12. doi: 10.18632/oncotarget.5658 (PMC4767472; doi:10.18632/oncotarget.5658)
Supplement: Supplementary file 1 [file oncotarget-06-42803-s001.pdf]

SUPPLEMENTARY TABLES

Supplementary Table S1: Baseline characteristics of patients from 1st data set

Patients' characteristics			Number (%)
**Age, years**	**Median (range)**		**59 (26–84)**
Gender	Male<br>Female		216 (62.6%)<br>129 (37.4%)
Baseline laboratory findings at diagnosis	Hemoglobin (g/dL)<br>MCV (fL)<br>Hemoglobin ≤11 g/dL, number (%)		13.18 ± 1.95<br>89.67 ± 6.81<br>49 (14.2%)
Disease	Gastric cancer<br>Colorectal cancer<br>Hepatocellular carcinoma<br>Lung cancer		101 (29.3%)<br>69 (20.0%)<br>23 (6.7%)<br>152 (44.1%)
First line treatment	Treatment modality other than surgery	Chemotherapy<br>Chemo-radiation<br>Target agents	215 (62.3%)<br>75 (21.7%)<br>55 (15.9%)
	Purpose of the treatment	Neoadjuvant<br>Adjuvant<br>Definitive<br>Palliative	6 (1.7%)<br>170 (49.3%)<br>15 (4.3%)<br>154 (44.6%)

Supplementary Table S2: Baseline characteristics of patients from 2nd data set

**Patients' characteristics**		**Number (%)**
**Age, years**	**Median (IQR, range)**	**58 (15, 27–82)**
Gender	Male<br>Female	27 (69.2%)<br>12 (30.8%)
Disease	Gastric cancer<br>Biliary tract cancer<br>Lung cancer<br>Lymphoma	11 (28.2%)<br>6 (15.4%)<br>18 (46.2%)<br>4 (10.3%)
Metastasis**	Yes<br>No	31 (88.6%)<br>4 (11.4%)
Chemotherapy within 6 months	Yes<br>No	26 (66.7%)<br>13 (33.3%)
Hemoglobin (g/dL)	Median (IQR)	9.5 (6.2)
MCV (fL)	Mean± SD	92.2 ± 1.3

Abbreviates: MCV, mean corpuscular volume; SD, standard deviation; IQR, interquartile range
**Lymphoma patients (n = 4) were excluded from the analysis

Supplementary Table S3: Biochemical parameters in cancer patients

				General population	Population in this study
Ferritin, ng/mL	Women	Mean	Premenopausal	53	224.7 ± 78.3*
			Post-menopausal	105	
		Range	Premenopausal	6–180	7.5–930.9
			Post-menopausal	8–350	
	Men	Mean		175	240.7 ± 36.1*
		Range		20–400	2.1–611.9
sTfR, ug/mL	Mean			0.854	1.03 ± 0.09
	Range			0.072–1.699	0.36–2.63
CRP, mg/L	Mean			–	14.73 ± 4.10
	Median			–	3.42 (13.95**)
	Range			0.068–8.2	0.01–100.00
Hepcidin, ng/mL	Mean			10.1	12.50 ± 1.78*
	Median			7.2	9.52 (16.18**, 1.19–50.33)

*Standard deviation (SD)
**Interquartile range (IQR)

Supplementary Table S4: Correlation between conventional iron parameters and cancer anemia related parameters

4–1

			**No anemia (n = 18)**	**Anemia (n = 21)**	***p*-value**
Biochemical parameters	Ferritin, ng/mL	Median (IQR)	166.7 ± 29.7	295.0 ± 55.5	**0.050**
	Iron	67.31+/−8.39<br>55.00 (74, 9–156)	89.5 (59, 37–139)	37 (62, 9–156)	**0.024**
	TIBC	275.0 (136, 164–435)	322.5 (114, 225–386)	266 (184, 164–435)	0.352
	TS	25.30 (20.2, 2.8–43.7)	29.6 (12.8, 15.6–40.6)	14.3 (24.7, 2.8–43.7)	0.069

4–2

		**Ferritin**		**sTfR**		**CRP**		**Hepcidin**		**Hemoglobin**	
		**r**	***p*-value**	**r**	***p*-value**	**r**	***p*-value**	**r**	***p*-value**	**r**	***p*-value**
Conventional iron parameters	Iron	–	0.951	–	0.063	−0.559	**0.002**	–	0.822	0.437	**0.018**
	TIBC	−0.698	**<0.000**	–	0.289	−0.497	**0.006**	−0.654	**<0.001**	–	0.696
	TS	–	0.179	–	0.187	−0.441	**0.017**	–	0.189	0.400	**0.031**
